# Aspirin vs. enoxaparin for thromboprophylaxis after total hip arthroplasty, total knee arthroplasty, or hip fracture surgery—a systematic review and meta-analysis

**DOI:** 10.3389/fmed.2026.1790739

**Published:** 2026-02-23

**Authors:** Abuduwupuer Haibier, Mailidan Maimaitiniyazi, Hang Lin, Wei Liu, Wencui Li

**Affiliations:** 1 Xinjiang Medical University, Urumqi, China; 2 Shenzhen Second People’s Hospital, Shenzhen, Guangdong, China

**Keywords:** knee arthroplasty, hip arthroplasty, ankle joint, aspirin, enoxaparin, hip fractures, meta-analysis, venous thromboembolism

## Abstract

**Objective:**

To systematically evaluate the efficacy and safety of aspirin vs. enoxaparin for the prevention of venous thromboembolism (VTE) following major orthopedic surgeries.

**Methods:**

We systematically searched PubMed, the Cochrane Library, Embase, China National Knowledge Infrastructure (CNKI), Wanfang, and CQVIP (VIP) databases from their inception until August 2025 for randomized controlled trials (RCTs) and cohort studies comparing aspirin with enoxaparin for thromboprophylaxis in patients undergoing major orthopedic surgeries (total hip/knee arthroplasty, hip fracture surgery). Two researchers independently performed literature screening, data extraction, and risk-of-bias assessment for the included studies. A meta-analysis was conducted using Review Manager (RevMan) version 5.3 software.

**Results:**

A total of studies involving 126,367 patients (43,441 in the aspirin group and 82,926 in the enoxaparin group) were included. Regarding efficacy, the initial meta-analysis showed no significant difference in the incidence of pulmonary embolism (PE; odds ratio [OR] = 1.14, 95% CI: 0.66–1.95) or deep vein thrombosis (DVT; primary analysis: Test for overall effect: Z = 0.43, *p* = 0.67). However, sensitivity analyses after removing key contributors to heterogeneity revealed that the incidence of DVT was significantly lower in the enoxaparin group (OR = 0.78, 95% CI: 0.64–0.96, *p* = 0.02), while the incidence of PE was significantly higher in the aspirin group (sensitivity analysis: Test for overall effect: Z = 2.30, *p* = 0.02). Regarding safety, the risks of both major bleeding (OR = 0.60, 95% CI: 0.40–0.89, *p* = 0.01) and minor bleeding (OR = 0.57, 95% CI: 0.49–0.66, *p* < 0.00001) were significantly lower in the aspirin group. No statistically significant differences were found between the two groups in terms of wound complications, 90-day all-cause mortality (primary analysis: OR = 0.81, 95% CI: 0.37–1.78, *p* = 0.60), or readmission rates.

**Conclusion:**

For patients undergoing major orthopedic surgery, aspirin is comparable to enoxaparin in preventing the primary efficacy outcomes of VTE (pulmonary embolism and deep vein thrombosis) but demonstrates a significant advantage in reducing the risk of minor bleeding. Aspirin represents an effective and safer prophylactic option, particularly for patients with higher bleeding risk profiles.

## 1 Introduction

Low-molecular-weight heparin (LMWH), particularly enoxaparin, has long been recommended as a first-line prophylactic agent for venous thromboembolism (VTE) following major orthopedic surgery by numerous international guidelines (1, 2), supported by its proven efficacy and extensive clinical evidence. Enoxaparin exerts its antithrombotic effect by potently and predictably inhibiting activated coagulation factor X (FXa) (3). However, its use is not without limitations. As a parenteral agent, it requires subcutaneous injections, which can be inconvenient for extended outpatient prophylaxis, potentially affecting adherence. More critically, its potent anticoagulant mechanism is associated with an increased risk of bleeding complications (4), which can worsen patient outcomes and increase healthcare utilization.

Aspirin, a well-established oral antiplatelet agent, has recently regained significant interest as an alternative. It works by irreversibly inhibiting platelet cyclooxygenase-1 (COX-1). Its advantages include convenient oral administration, significant cost-effectiveness, and a familiar safety profile. Although earlier studies suggested its efficacy was inferior to LMWH (5), recent large-scale, high-quality randomized controlled trials (RCTs) have provided compelling new evidence, indicating that for patients undergoing total hip or knee arthroplasty, aspirin is non-inferior to enoxaparin in preventing symptomatic VTE and may be superior for certain safety endpoints (2, 6, 7). This evidence has influenced clinical guidelines, such as those from the American Academy of Orthopedic Surgeons (AAOS), which now list aspirin as a viable option (8).

Despite this, considerable academic debate persists regarding the comparative merits of aspirin vs. enoxaparin. Findings from existing studies remain inconsistent: some meta-analyses support aspirin’s comparable efficacy and superior safety profile (9, 10), while others maintain that enoxaparin’s potent anticoagulant effect remains essential for high-risk patients (11). These discrepancies may stem from limitations of individual RCTs, including small sample sizes, heterogeneous patient populations, varying follow-up durations, and non-uniform outcome definitions.

The compelling evidence of aspirin’s potential non-inferiority or superiority in safety endpoints from these recent high-quality RCTs necessitates an updated, comprehensive synthesis of the available data. Therefore, this study aims to systematically search domestic and international databases to identify studies that compare aspirin and enoxaparin for VTE prophylaxis in patients undergoing major orthopedic surgery. After rigorous quality assessment, we will conduct a meta-analysis to systematically compare the two agents regarding their efficacy in preventing pulmonary embolism (PE) and deep vein thrombosis (DVT) and their safety profiles concerning major and minor bleeding, wound complications, mortality, and readmission rates. The objective is to provide higher-level evidence to inform clinical practice and personalized anticoagulation decisions.

## 2 Data and methods

### 2.1 Literature search strategy

#### 2.1.1 Search personnel

The first author conducted the literature search.

#### 2.1.2 Databases

The following electronic databases were searched via computer: PubMed, Cochrane Library, Embase, China National Knowledge Infrastructure (CNKI), Wanfang database, and CQVIP (VIP) database (CQVIP). This systematic review and meta-analysis were designed and reported in accordance with the Preferred Reporting Items for Systematic Reviews and Meta-Analyses (PRISMA) guidelines (12).

#### 2.1.3 Search terms

“Aspirin, acetylsalicylic acid, orthopedic major surgery, orthopedic surgery, enoxaparinn, deep vein thrombosis, DVT.”

#### 2.1.4 Timeframe

Searches were conducted from the inception of each database up to August 2025 for studies comparing aspirin and low-molecular-weight heparin in the prevention of deep vein thrombosis after major orthopedic surgery.

#### 2.1.5 Search strategy

The specific search strategy for the PubMed database is detailed in Table 1. Additionally, the references of included studies were manually reviewed to identify further potentially relevant publications.

**Table 1:** Search strategy for the PubMed database.

#1	(“Arthroplasty, Replacement, Hip”[Mesh] OR “Arthroplasty, Replacement, Knee”[Mesh] OR “Hip Fractures/surgery”[Mesh] OR ((total hip replace[Title/Abstract] OR total hip arthroplast[Title/Abstract] OR THA[Title/Abstract] OR THR[Title/Abstract]) OR (total knee replace[Title/Abstract] OR total knee arthroplast[Title/Abstract] OR TKA[Title/Abstract] OR TKR[Title/Abstract]) OR (hip fracture[Title/Abstract] OR femoral neck fracture[Title/Abstract])))
#2	(“Aspirin”[Mesh] OR “Platelet Aggregation Inhibitors”[Mesh] OR aspirin[Title/Abstract] OR acetylsalicylic acid[Title/Abstract] OR ASA[Title/Abstract])
#3	(“Enoxaparin”[Mesh] OR “Heparin, Low-Molecular-Weight”[Mesh] OR enoxaparin[Title/Abstract] OR Lovenox[Title/Abstract] OR “low molecular weight heparin”[Title/Abstract] OR LMWH[Title/Abstract])
#4	#1 AND (#2 OR #3)

### 2.2 Inclusion and exclusion criteria

#### 2.2.1 Inclusion criteria

(1) Population: Patients undergoing major orthopedic surgery (including hip/knee arthroplasty or hip fracture surgery) requiring postoperative anticoagulant therapy. (2) Study Type: RCTs or retrospective cohort studies. (3) Interventions: The aspirin group received perioperative aspirin for anticoagulation, while the enoxaparin group received perioperative enoxaparin for anticoagulation. (4) Outcome Measures: Comparative data on the incidence of pulmonary embolism (PE), DVT, major bleeding events (defined as bleeding leading to impaired organ/limb/muscle function requiring reoperation), minor bleeding events (e.g., ecchymosis, hematoma, and wound bleeding), wound complications (e.g., infection and drainage), 90-day all-cause mortality, and readmission rates.

#### 2.2.2 Exclusion criteria

(1) Studies with fewer than 20 cases. (2) Reviews, case reports, or conference abstracts. (3) Literature with incomplete or unavailable original data. (4) Duplicate publications.

### 2.3 Data extraction

Literature management software (EndNote X9, Thomson Scientific) was used to collate the search results and remove duplicates. Two investigators independently screened the titles and abstracts of the deduplicated records, excluding those clearly irrelevant. The full texts of the remaining articles were then retrieved and assessed for eligibility based on the predefined inclusion and exclusion criteria. The quality of the finally included studies was assessed, and relevant data were independently extracted by the same two researchers. To ensure the consistency and reliability of the screening and data extraction process, inter-rater agreement was calculated using Cohen’s kappa statistic at both the title/abstract screening and full-text assessment stages, yielding kappa values of 0.85 and 0.88, respectively, indicating excellent agreement. The extracted data were summarized and cross-checked. Any discrepancies in the data extraction process were resolved through consultation with a third independent researcher. If necessary, corresponding authors were contacted to obtain complete original data.

### 2.4 Literature quality assessment

The quality of all included studies was independently assessed by two reviewers. The Newcastle–Ottawa Scale (NOS) was used to evaluate the quality of cohort studies and case–control studies (13). In the NOS assessment, studies are scored out of a maximum of 9 points. Those with a score ≥7 are considered high quality, scores of 5–6 indicate moderate quality, and scores <5 are deemed low quality. For RCTs, the Cochrane risk-of-bias tool in Review Manager (RevMan) version 5.3 was utilized (14). The assessment focused on seven key domains: random sequence generation, allocation concealment, blinding of participants and personnel, blinding of outcome assessment, incomplete outcome data, selective reporting, and other potential sources of bias. A risk-of-bias graph was generated accordingly. Any disagreements in the quality assessments were resolved through discussion with a third independent reviewer. The inter-rater reliability for the risk-of-bias assessments, as measured by Cohen’s kappa, was 0.82, indicating strong agreement between the two reviewers.

### 2.5 Outcome measures

The extracted data encompassed the following two primary categories: (1) Baseline characteristics, which included the first author, year of publication, country/region, study design, sample size, patient age, type of surgery, specific intervention regimens, and follow-up duration; and (2) Outcomes of interest, specifically the incidence rates of PE, DVT, major bleeding (defined as bleeding leading to impaired organ/limb/muscle function requiring re-operation), minor bleeding (e.g., ecchymosis, hematoma, and wound bleeding), wound complications (e.g., infection and drainage), 90-day all-cause mortality, and readmission rates. It is acknowledged that the diagnostic criteria for VTE events (DVT and PE) varied across the included studies. The outcome data for VTE included both symptomatic events confirmed by objective imaging (such as compression ultrasonography, venography, or computed tomography pulmonary angiography) and, in some studies, events detected through systematic screening imaging protocols. Data regarding the specific diagnostic methods employed in each included study were extracted and considered during the analysis and interpretation of the results.

### 2.6 Statistical analysis

Meta-analysis was performed using RevMan version 5.3 software.^1^ For dichotomous data, the odds ratio (OR) was used as the effect measure. All effect measures are reported with their 95% confidence intervals (CIs). Heterogeneity was assessed using the I^2^ statistic and *p*-value: if *p* > 0.1 and I^2^ < 50%, heterogeneity among studies was considered low, and a fixed-effect model was applied; if *p* ≤ 0.1 and *I*^2^ ≥ 50%, a random-effects model was used, and subgroup or sensitivity analyses were conducted to explore sources of heterogeneity. Sensitivity analysis was performed by sequentially excluding individual studies to assess the impact of each study on the overall effect size. Publication bias was evaluated using funnel plots. A significance level of *p* ≤ 0.05 was set for all statistical analyses. We recognized that variations in the dosage (e.g., aspirin 81 mg vs. 325 mg; enoxaparin 40 mg once daily vs. 30 mg twice daily) and duration of prophylaxis among the included studies represent important sources of clinical heterogeneity. *A priori*, we planned to explore the potential impact of these factors through subgroup analyses if sufficient data were available.

### 2.7 Evidence quality assessment (grading of recommendations, assessment, development and evaluation [GRADE])

The quality of evidence for each primary outcome (incidence of pulmonary embolism, deep vein thrombosis, major bleeding, and minor bleeding) was assessed using the GRADE (15) (Grading of Recommendations, Assessment, Development, and Evaluation) framework. Evidence from randomized controlled trials (RCTs) initially rated as high quality may be downgraded for five factors: risk of bias, inconsistency, indirectness, imprecision, and publication bias. Evidence from observational (cohort) studies began as low quality and could be further downgraded for the same reasons, or upgraded for factors such as a large effect size or a dose–response gradient. The evidence quality for each outcome was categorized into one of four levels: high, moderate, low, or very low. This assessment was performed independently by two reviewers, with any disagreements resolved by consensus.

## 3 Results

### 3.1 Literature search results and literature screening flow

**Figure 1:**
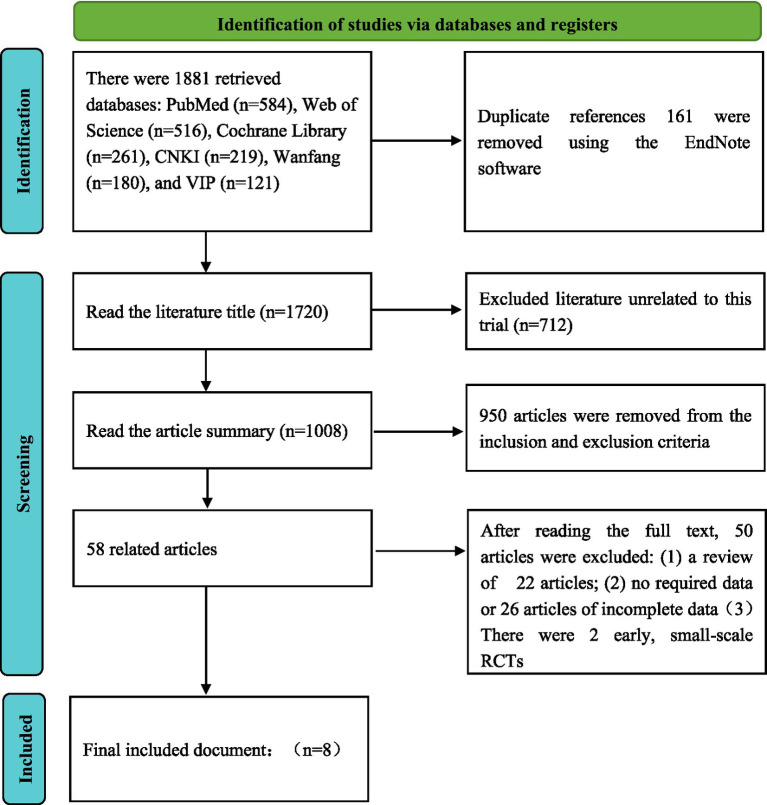
Literature screening process and results.

A total of 1,881 records were initially identified through database searches. After removing duplicates and a rigorous screening process based on titles, abstracts, and full texts, 8 studies meeting the predefined criteria were included for the final analysis [comprising 4 RCTs (6, 11, 16, 17) and 4 cohort studies (7, 18–20)]. The study selection process is detailed in Figure 1. These studies comprised a total of 126,367 patients, with 43,441 patients in the aspirin group and 82,926 patients in the enoxaparin group. See Table 2 for the basic characteristics of the included studies.

**Table 2:** Basic characteristics of the eight studies included.

Author (year)	Country	Study type	Surgery type	Sample size		Age		BMI (kg/m^2^)		Intervention		Follow-up	Outcome measures
				Aspirin	Enoxaparin	Aspirin	Enoxaparin	Aspirin	Enoxaparin	Aspirin	Enoxaparin		
Sidhu, 2022 (11)	Australia	RCT	TKA + THA	14,156	9,302	69 (62–77)	70 (62–77)	30.0 (26.2–34.6)	29.9 (26.1–34.5)	100 mg/day for 35 days (oral)	40 mg/day for 35 days (SC)	90 days	⑥
Cortes, 2021 (16)	Mexico	RCT	TKA	188	214	70.33	71.31	NS	NS	100 mg/day for 30 days (Oral)	40–60 mg/day for 30 days (SC)	90 days	①②③④⑤
CRISTAL study group, 2024 (6)	Australia	RCT	TKA + THA	6,901	4,827	68	69	30.5	30.4	100 mg once daily (oral); Duration: Hip 35 days; Knee 14 days	40 mg once daily (SC); Duration: Hip 35 days, Knee 14 days	90 days	①②③⑤⑥⑦
Flores, 2024 (17)	Mexico	RCT	TKA + THA	30	30	68.5 ± 8.6	68.5 ± 8.6	28.8 ± 4.5	28.8 ± 4.5	100 mg once daily for 5 weeks (oral)	40 mg once daily for 5 weeks (SC)	90 days	②④⑤
Lindquist, 2018 (18)	USA	CC	TKA + THA	366	438	65.8	66.7	NS	NS	325 mg twice daily (oral); Duration: TKA 12 days, THA 35 days	30 mg (twice daily) or 40 mg (once daily) (SC); Duration: TKA 12 days, THA 35 days	30 days	②③④⑤⑦
Liu, 2024 (7)	USA	CC	THA + TKA	4,293	5,728	69 ± 10.4	69 ± 10.4	NS	NS	81 mg or 325 mg twice daily (oral)	30 mg (twice daily) or 40 mg (once daily) (SC)	90 days	①②③⑤⑥⑦
Cheallaigh, 2020 (19)	Ireland	CC	TKA + THA	3,460	961	NS	NS	NS	NS	150 mg once daily for 28 days (oral)	40 mg once daily for 28 days (SC)	180 days	①②
Paula, 2021 (20)	USA	CC	TKA + THA	14,047	61,426	66.77 ± 10.14	69.59 ± 11.52	29.59 ± 4.96	28.09 ± 5.49	650 mg/day for 40 days (oral)	40 mg/day for 40 days (SC)	40 days	②③④⑥

NS, not specified; BMI, body mass index; RCT, randomized controlled trial; CC, case–control; TKA, total knee arthroplasty; THA, total hip arthroplasty; SC, subcutaneously. Outcome Measures: ① Incidence of pulmonary embolism; ② Incidence of deep vein thrombosis (DVT); ③ Incidence of major bleeding events (defined as bleeding leading to reoperation due to impairment of organ, limb, or muscle function); ④ Incidence of minor bleeding events (including subcutaneous ecchymosis, hematoma, or wound bleeding); ⑤ Incidence of wound complications (including surgical site infection and wound drainage); ⑥ All-cause mortality within 90 days postoperatively; ⑦ 90-day readmission rate.

### 3.2 Quality assessment of included studies

#### 3.2.1 Randomized controlled trials

**Figure 2:**
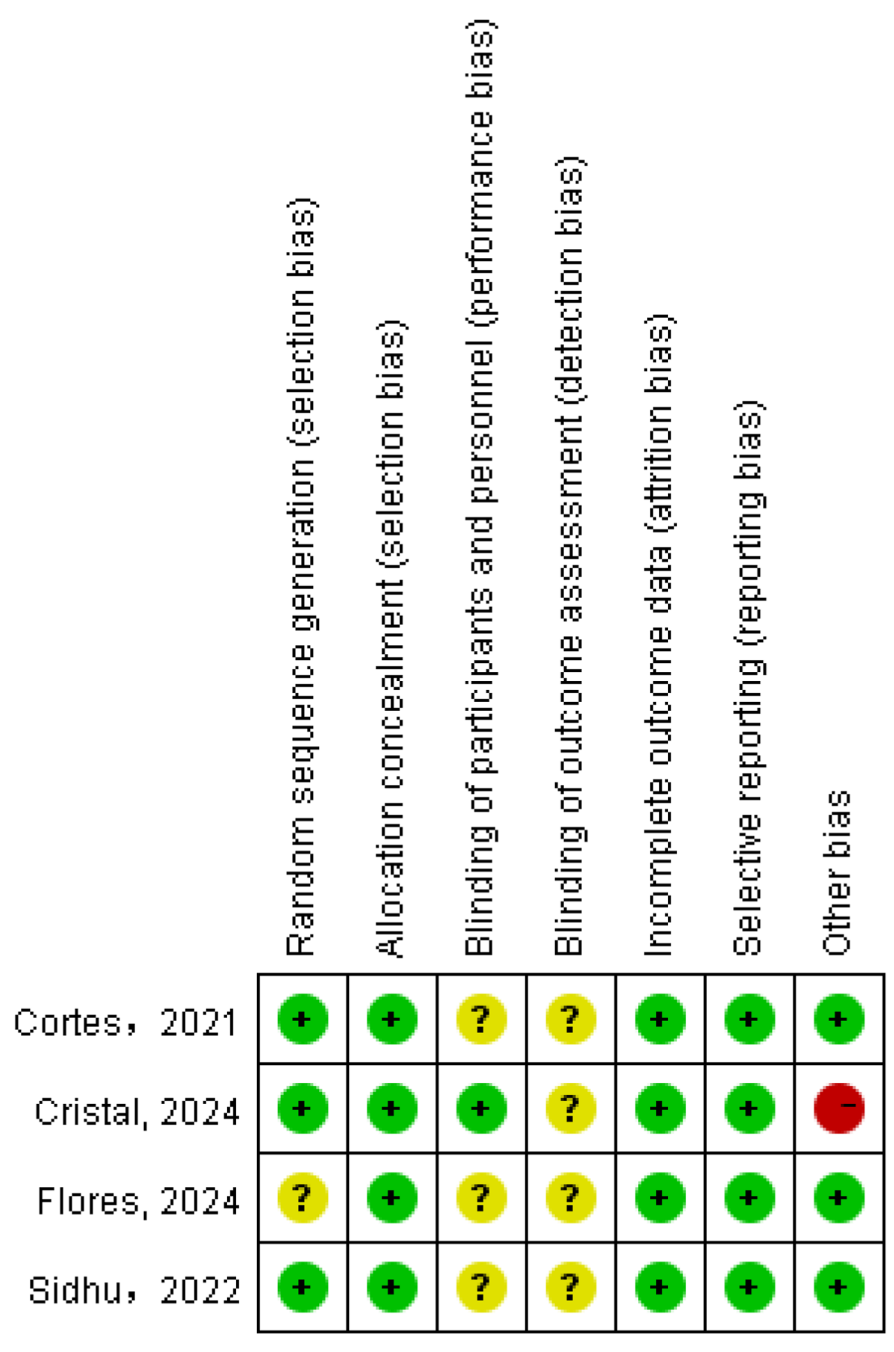
Evaluation of the methodological quality of the included studies. “+” (green) indicates low risk of bias; “?” (yellow) indicates unclear risk of bias.

All included studies provided detailed descriptions of consistent baseline characteristics across comparison groups. Regarding risk-of-bias assessment among the RCTs included in this analysis, the evaluations for individual studies are presented in Figure 2. As illustrated, the trials by Cortes (16), CRISTAL study group (6), and Sidhu (11) were predominantly assessed as having a low risk of bias across most key domains, including random sequence generation. The study by Flores (17) was assessed as having an unclear risk across several domains.

**Figure 3:**
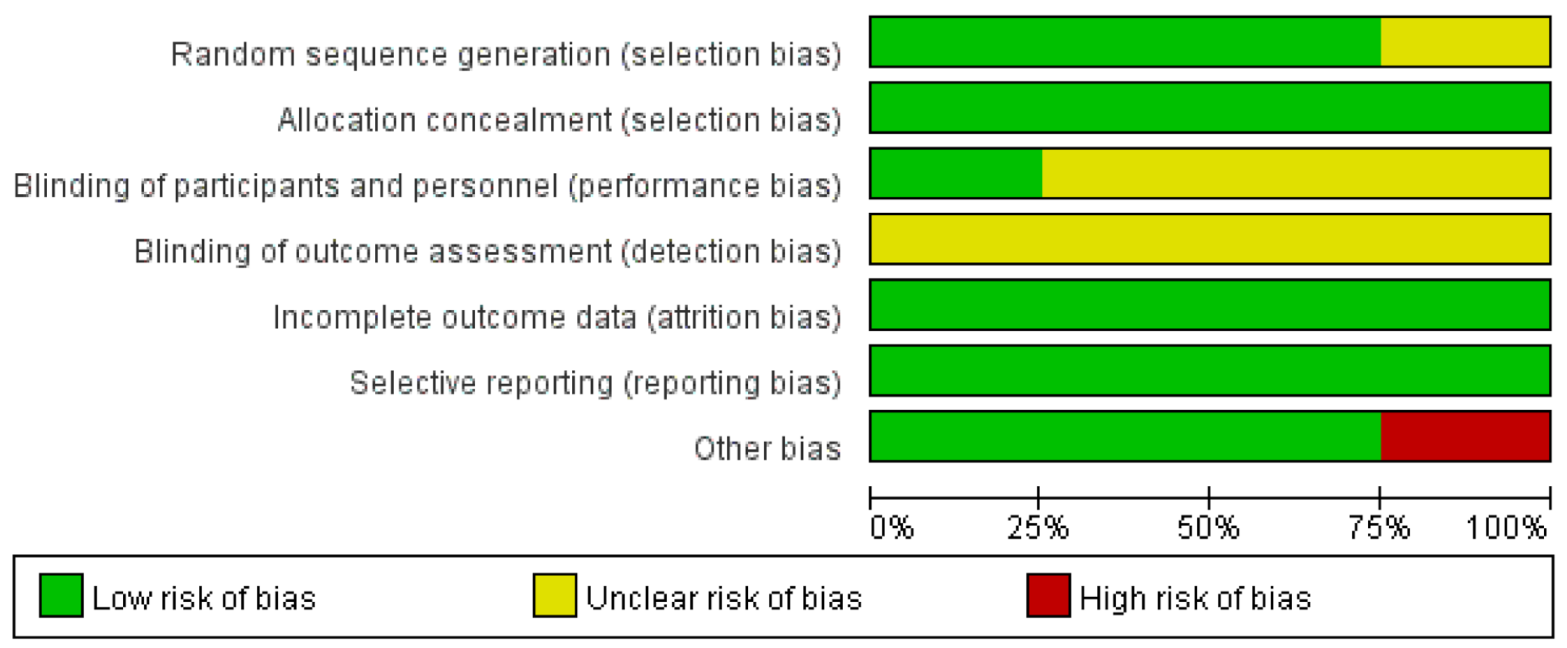
Percentage of risk of bias. Green indicates low risk of bias; yellow indicates unclear risk of bias; red indicates high risk of bias.

Figure 3 summarizes the methodological quality across all assessed studies. It indicates that the domains of allocation concealment and blinding of outcome assessment were judged to have a low risk of bias in all studies where applicable. The domain of random sequence generation also showed a low risk of bias in the majority of studies. Overall, the methodological quality of the included RCTs was considered acceptable for this meta-analysis.

#### 3.2.2 Retrospective cohort studies

The methodological quality of the four included retrospective cohort studies (7, 18–20) was assessed using the Newcastle–Ottawa Scale (NOS). The results indicated that all studies were of high quality. Specifically, the studies by Lindquist (18) and Paula (20) achieved the maximum score of 8 points, demonstrating excellence in both cohort selection and comparability. The studies by Liu (7) and Cheallaigh (19) each received a score of 7 points, with the primary point of differentiation being the comparability of groups. These assessment results robustly affirm the high methodological rigor and internal validity of the evidence synthesized in this systematic review. Detailed ratings are presented in Table 3.

**Table 3:** Quality assessment of retrospective cohort studies (NOS grade).

First author/year	Selection (stars)	Comparability (stars)	Outcome (stars)	Total score (stars)	Quality rating
Lindquist, 2018 (18)	3	3	2	8	High
Liu, 2024 (7)	3	2	2	7	High
Cheallaigh, 2020 (19)	3	2	2	7	High
Paula, 2021 (20)	3	3	2	8	High

The Newcastle–Ottawa Scale (NOS) has a maximum score of 9. A score ≥7 indicates high quality, a score of 5–6 indicates moderate quality, and a score <5 indicates low quality.

### 3.3 Meta-analysis results

#### 3.3.1 Incidence of pulmonary embolism

**Figure 4:**
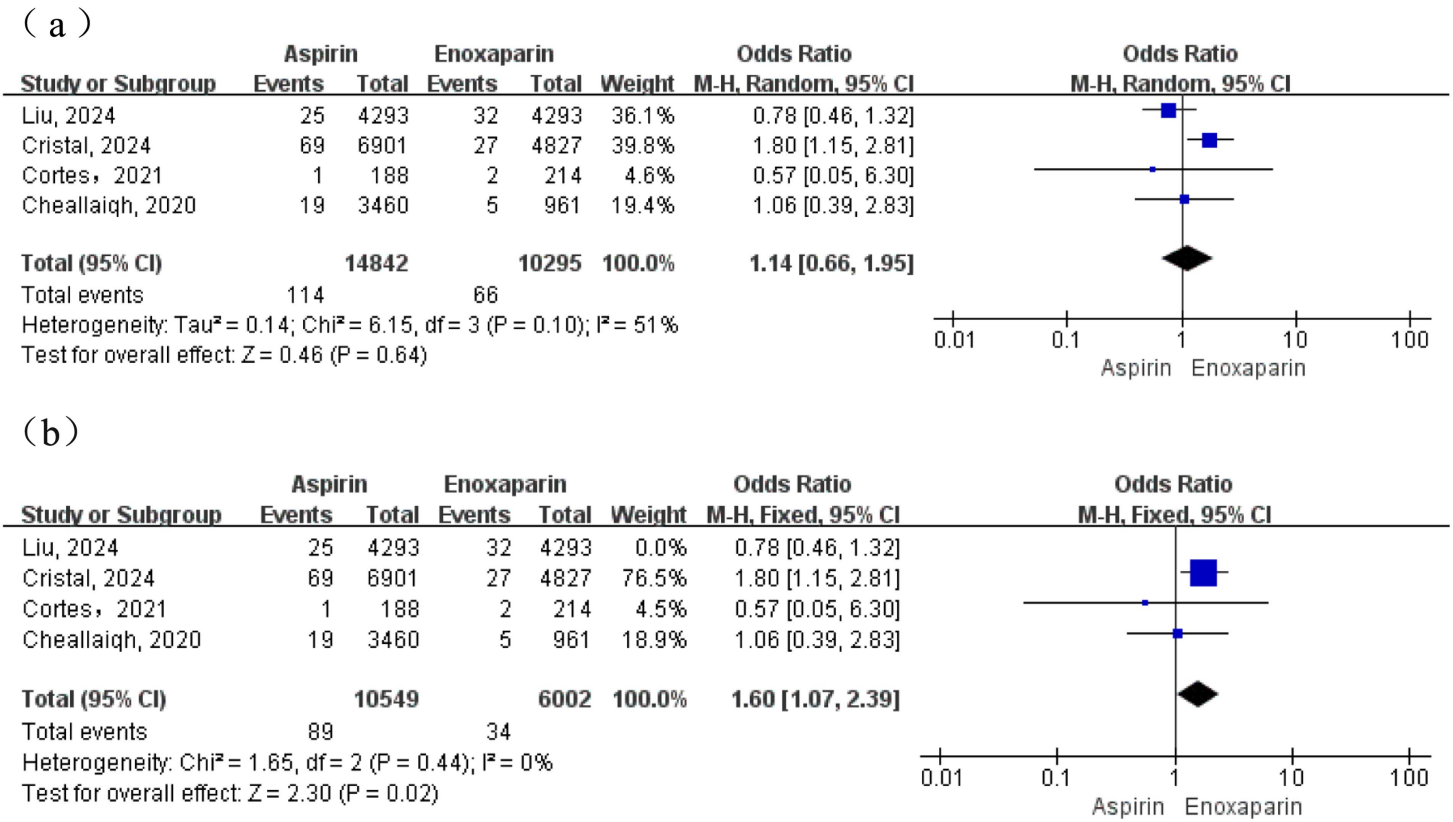
**(a)** Forest plot of the meta-analysis comparing the incidence of pulmonary embolism between the two groups after treatment; **(b)** Sensitivity analysis.

A total of four studies were included for this outcome. Moderate heterogeneity was observed among the studies (I^2^ = 51%; *p* = 0.10); therefore, a random-effects model was initially used for the meta-analysis. The results demonstrated no statistically significant difference in the incidence of pulmonary embolism between the aspirin and enoxaparin groups (OR = 1.14, 95% CI: 0.66–1.95). Subsequently, in an analysis employing a fixed-effects model after excluding a contributing study, heterogeneity was substantially reduced (I^2^ = 0%, *p* = 0.44). This analysis revealed that the incidence of pulmonary embolism was significantly higher in the aspirin group compared to the enoxaparin group (test for overall effect: Z = 2.30, *p* = 0.02) (Figure 4).

#### 3.3.2 Incidence of deep vein thrombosis

**Figure 5:**
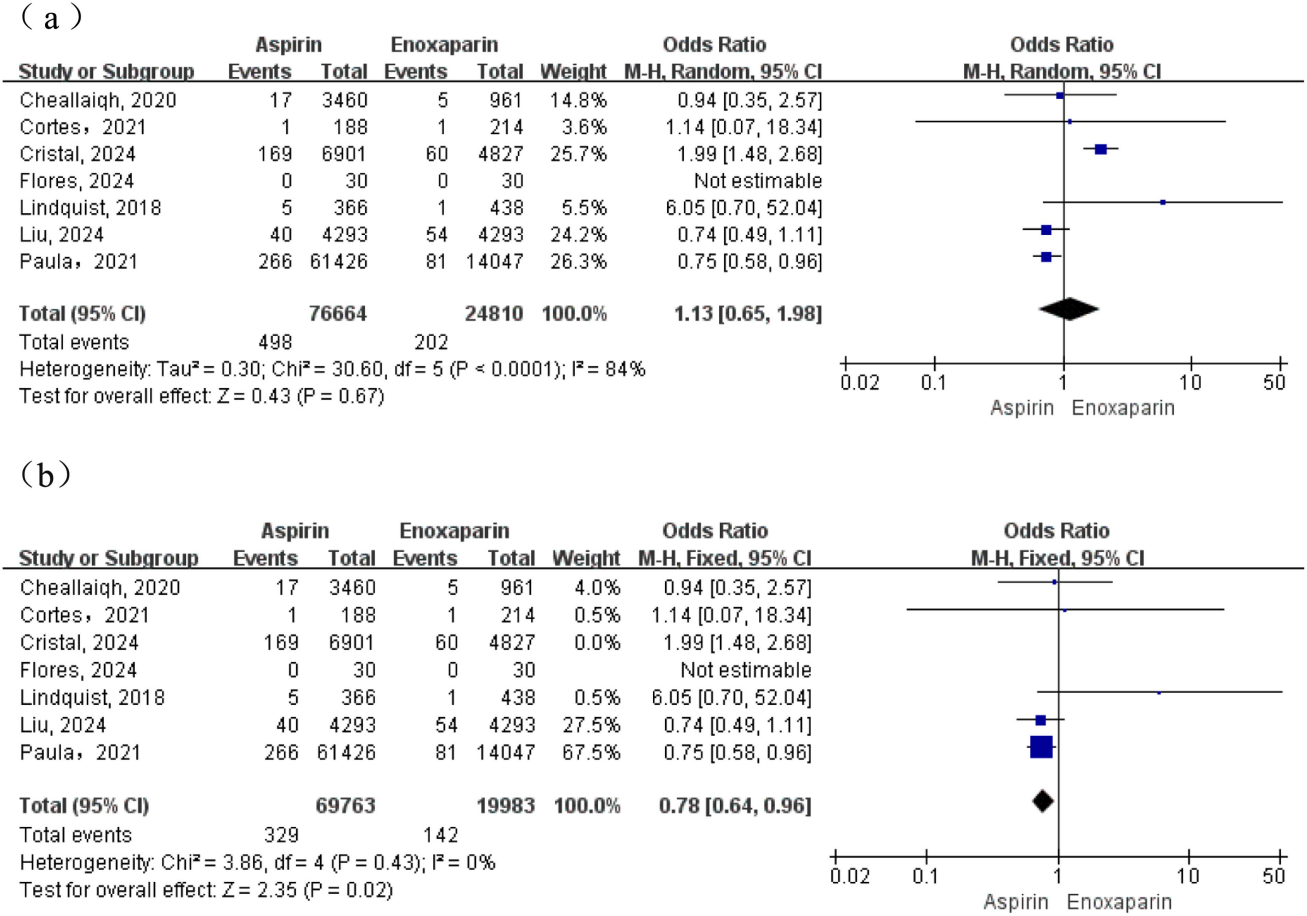
**(a)** Forest plot of the meta-analysis comparing the incidence of deep vein thrombosis between the two groups after treatment; **(b)** Sensitivity analysis.

A total of seven studies were included for this outcome. Substantial heterogeneity was observed among the studies (I^2^ = 84%; *p* < 0.0001); therefore, a random-effects model was used for the primary meta-analysis. The results demonstrated no statistically significant difference in the incidence of DVT between the aspirin and enoxaparin groups (test for overall effect: Z = 0.43, *p* = 0.67), suggesting comparable efficacy in preventing DVT (Figure 5a). Given the significant heterogeneity, a sensitivity analysis was performed. The study by the CRISTAL study group (6) was excluded as it was identified as a primary contributor to heterogeneity. This decision was based not only on its statistical influence but also on key clinical and methodological features: it was the largest RCT, and although it did not mandate universal systematic screening, its open-label design led to a higher proportion of patients in the aspirin group undergoing ultrasound surveillance. This likely resulted in increased detection of asymptomatic distal DVTs compared to studies that relied solely on clinically suspected, symptomatic VTE, representing a potential source of detection bias. The post-sensitivity analysis indicated that heterogeneity was substantially reduced (I^2^ = 0%, *p* = 0.43), allowing the use of a fixed-effects model. This adjusted analysis revealed that the incidence of DVT was significantly lower in the enoxaparin group compared to the aspirin group (OR = 0.78, 95% CI: 0.64–0.96, *p* = 0.02), as shown in Figure 5b.

#### 3.3.3 Incidence of major bleeding events

**Figure 6:**
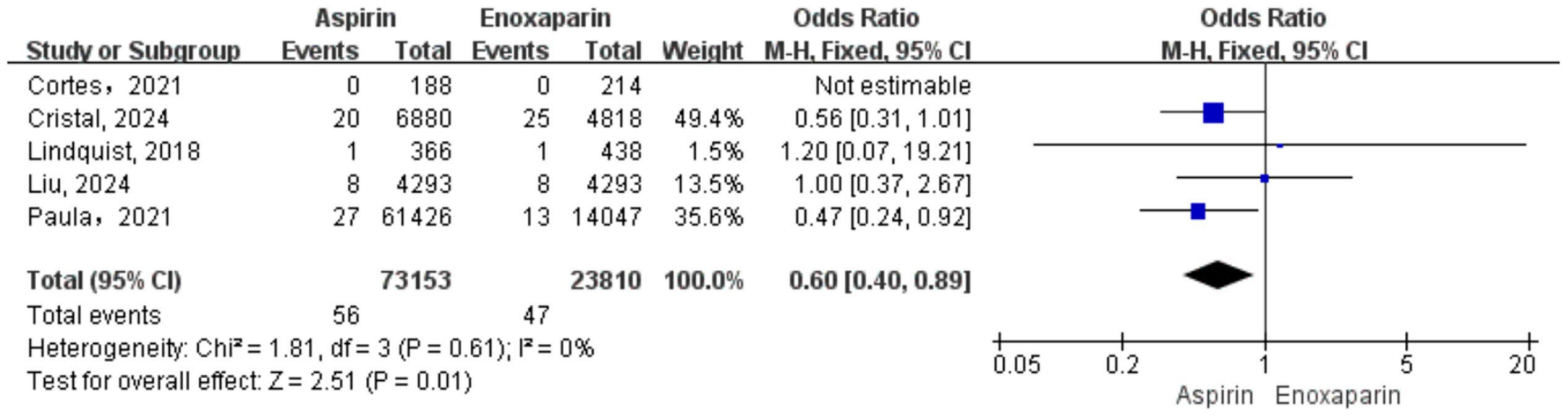
Forest plot of the meta-analysis comparing the incidence of major bleeding events between the two groups after treatment.

A total of five studies were included for the analysis of major bleeding events. No significant heterogeneity was observed among the included studies (I^2^ = 0%, *p* = 0.61), a fixed-effects model was employed for the meta-analysis. The results demonstrated that the incidence of major bleeding events was significantly lower in the aspirin group compared to the enoxaparin group (OR = 0.60, 95% CI: 0.40–0.89; Test for overall effect: Z = 2.51, *p* = 0.01), indicating a reduced risk of major bleeding associated with aspirin prophylaxis in this analysis. The forest plot for this analysis is presented in Figure 6.

#### 3.3.4 Incidence of minor bleeding events

**Figure 7:**
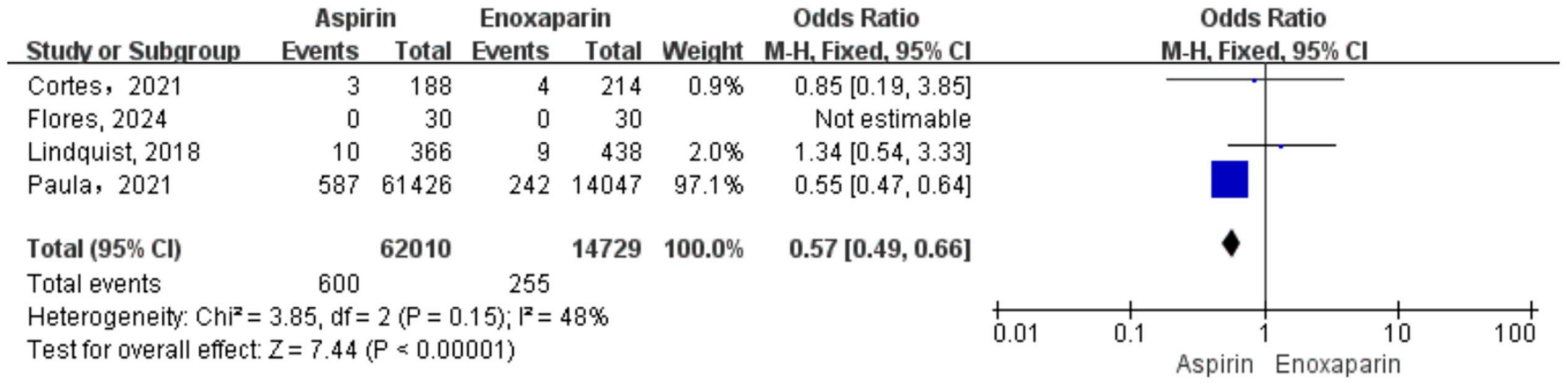
Forest plot of the meta-analysis comparing the incidence of minor bleeding events between the two groups after treatment.

A total of four studies were included in the analysis of minor bleeding events. Among them, one study (17) with zero events in both groups was not estimable and therefore did not contribute to the pooled analysis. Moderate heterogeneity was observed among the remaining studies (I^2^ = 48%, *p* = 0.15), thus a fixed-effects model was applied. The meta-analysis demonstrated a statistically significant difference in the incidence of minor bleeding events between the aspirin and enoxaparin groups (OR = 0.57, 95% CI: 0.49–0.66; Test for overall effect: Z = 7.44, *p* < 0.00001). This result indicates that the risk of minor bleeding was significantly lower in the aspirin group compared to the enoxaparin group following major orthopedic surgery, as detailed in Figure 7.

#### 3.3.5 Incidence of wound complications

**Figure 8:**
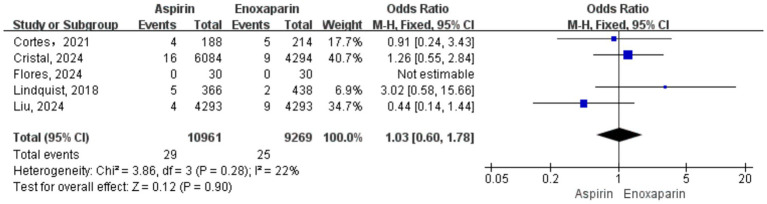
Forest plot of the meta-analysis comparing the incidence of wound complications between the two groups after treatment.

Five studies were included for the analysis of wound complications. Given the low heterogeneity among the studies (*p* = 0.28, I^2^ = 22%), a fixed-effects model was utilized for the meta-analysis. The results showed no statistically significant difference in the incidence of wound complications between the aspirin and enoxaparin groups (OR = 1.03, 95% CI: 0.60–1.78, *p* = 0.90), suggesting no significant difference in the risk of wound complications between the two prophylactic strategies. The forest plot for this outcome is presented in Figure 8.

#### 3.3.6 All-cause mortality within 90 days postoperative

**Figure 9:**
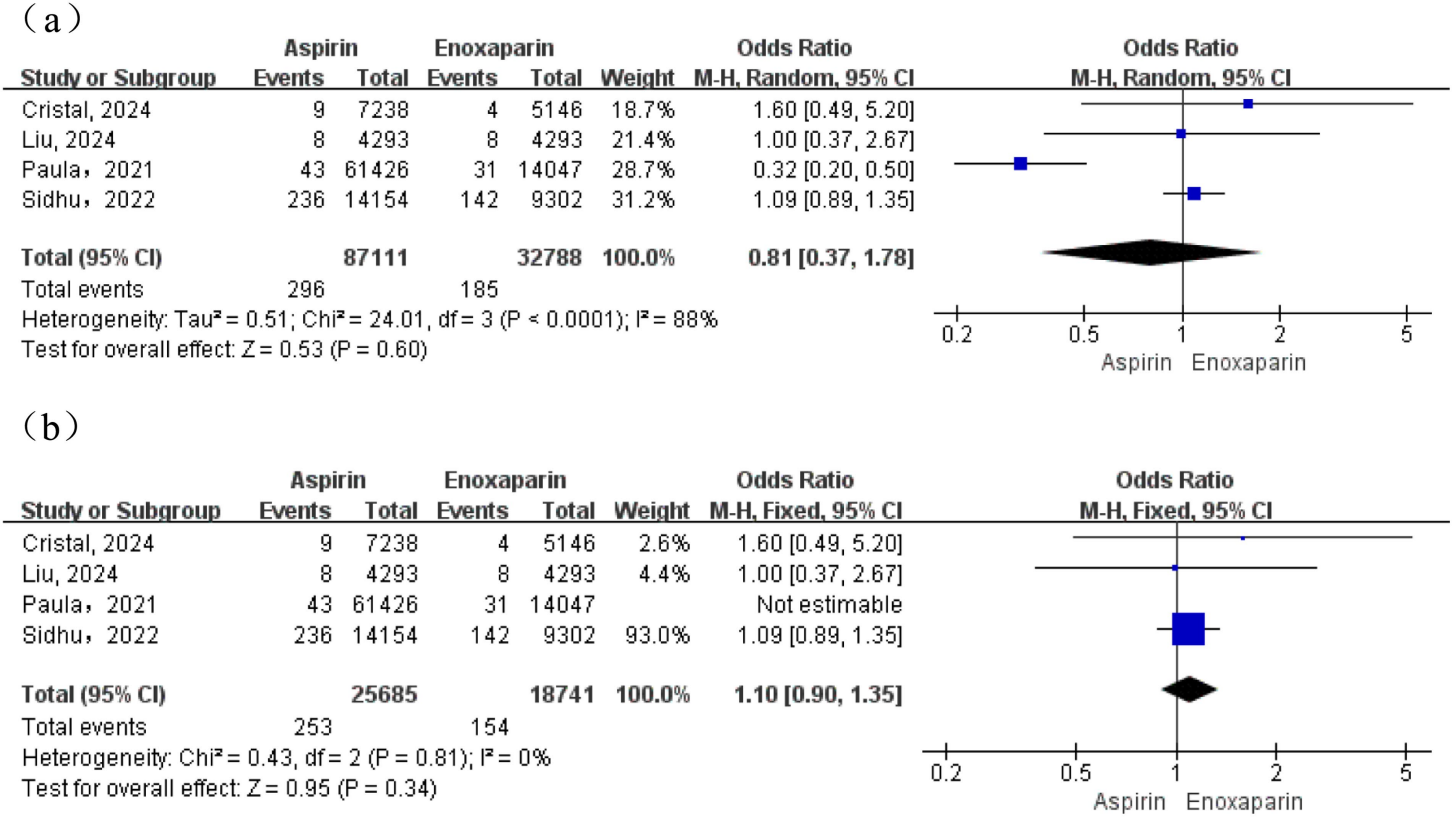
**(a)** Forest plot of the meta-analysis comparing the 90-day all-cause mortality between the two groups; **(b)** Sensitivity analysis.

A total of four studies were included in the analysis of 90-day all-cause mortality. The initial assessment revealed substantial heterogeneity among studies (I^2^ = 88%; *p* < 0.0001), warranting a random-effects model for the primary meta-analysis. The results demonstrated no statistically significant difference in mortality between the aspirin and enoxaparin groups (OR = 0.81, 95% CI: 0.37–1.78; test for overall effect: Z = 0.53, *p* = 0.60), as shown in Figure 9a. Given the substantial heterogeneity observed, a sensitivity analysis was conducted. The study by Paula (20) was excluded as it was the main source of heterogeneity. This was justified by its distinct characteristics: it was a very large retrospective cohort study (*n* = 75,473) that utilized a North American administrative database, involved a different aspirin dosing regimen (650 mg daily), and had a shorter, unique follow-up period of 40 days compared to the 90-day follow-up in the other studies. The post-sensitivity analysis indicated that heterogeneity was substantially reduced (I^2^ = 0%, *p* = 0.81), thereby allowing the use of a fixed-effects model. This adjusted analysis continued to show no statistically significant difference in mortality between the two groups (OR = 1.10, 95% CI: 0.90–1.35; Test for overall effect: Z = 0.95, *p* = 0.34), as presented in Figure 9b.

#### 3.3.7 Readmission rates

**Figure 10:**
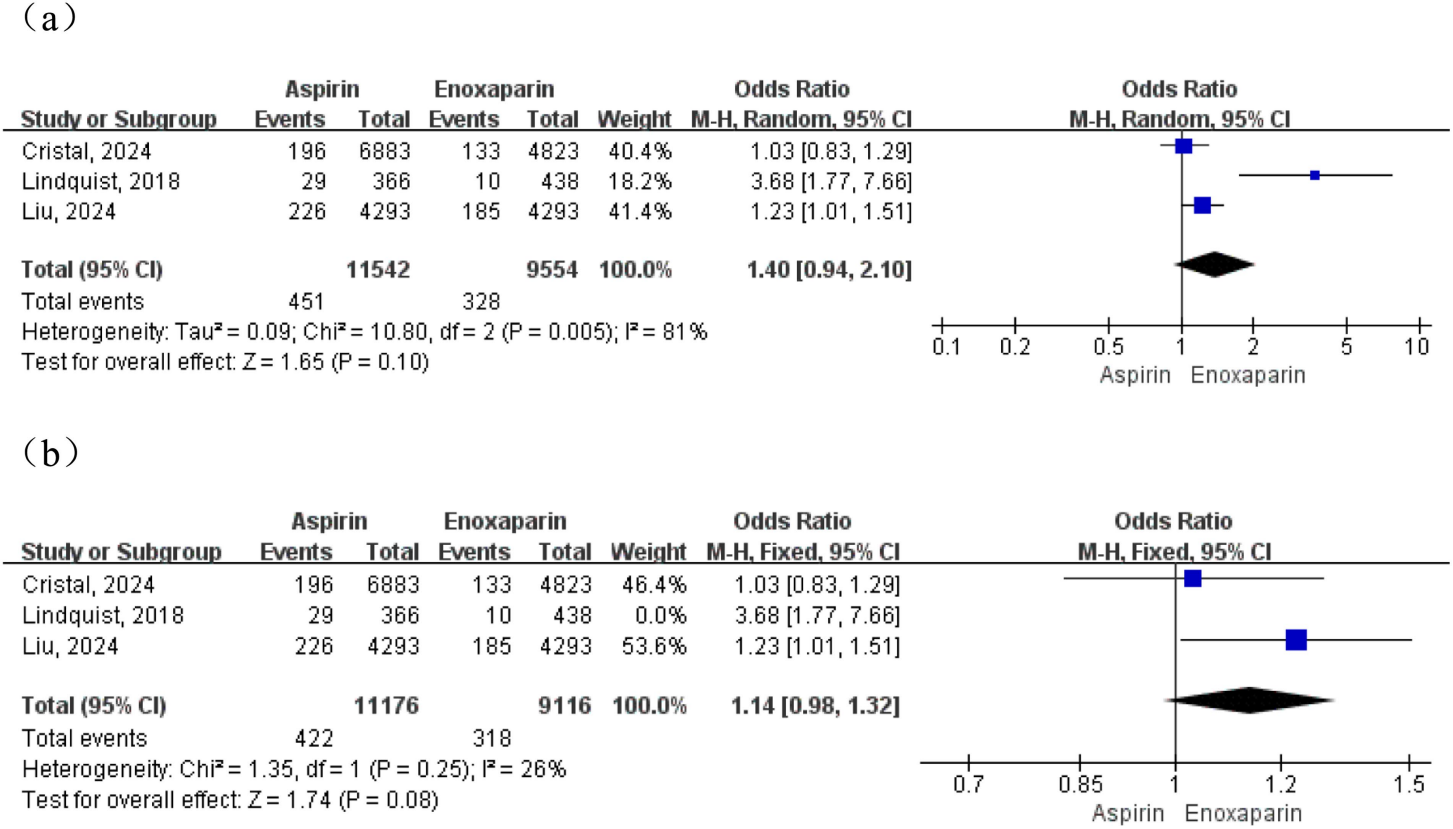
**(a)** Meta-analysis of readmission rates after treatment in two groups; **(b)** Sensitivity analysis.

Three studies reported data on postoperative readmission rates. The initial analysis indicated substantial heterogeneity among the studies (*p* = 0.005, I^2^ = 81%); therefore, a random-effects model was employed for the primary meta-analysis. The results demonstrated no statistically significant difference in readmission rates between the aspirin and enoxaparin groups (OR = 1.40, 95% CI: 0.94–2.10, *p* = 0.10), suggesting a comparable risk of postoperative readmission between the two strategies, as shown in Figure 10a. Given the substantial heterogeneity, a sensitivity analysis was performed by excluding one study (18). The post-sensitivity analysis showed a significant reduction in heterogeneity (I^2^ = 26%, *p* = 0.25), permitting the use of a fixed-effects model. This adjusted analysis continued to demonstrate no statistically significant difference in readmission rates between the groups (OR = 1.14, 95% CI: 0.98–1.32, *p* = 0.08), as shown in Figure 10b.

### 3.4 Quality of evidence

The GRADE assessments for the key efficacy and safety outcomes are summarized in Table 4. The quality of evidence was rated as moderate for the outcomes of pulmonary embolism and major bleeding, indicating that we are moderately confident in the effect estimates, and the true effect is likely to be close to the estimate, but there is a possibility that it is substantially different. The evidence for deep vein thrombosis was rated as low quality, and for minor bleeding as moderate quality. Downgrading was primarily due to inconsistency (substantial unexplained heterogeneity) and imprecision (wide confidence intervals crossing the line of no effect or minimal important difference).

**Table 4:** Summary of findings and quality of evidence (GRADE) for main outcomes.

Outcomes	Relative effect (95% CI)	Anticipated absolute effects^a^ (95% CI)	Number of participants (studies)	Quality of evidence (GRADE)	Comments
Pulmonary embolism Follow-up: 90 days	OR 1.14 (0.66–1.95)	12 per 10,000 (to be updated)	25137 (4 studies)	⊕⊕◯◯ Low	Downgraded two levels: one for serious inconsistency (moderate heterogeneity, I^2^ = 51%) and one for imprecision (wide CI crossing the line of no effect).
Deep vein thrombosis Follow-up: 90 days	Primary analysis: Test for overall effect: Z = 0.43 (*p* = 0.67). (Sensitivity analysis OR = 0.78 [0.64–0.96])	16 per 10,000 (to be updated)	101474 (7 studies)	⊕◯◯◯ Very low	Downgraded three levels: one for serious inconsistency (considerable heterogeneity, I^2^ = 84%), one for serious imprecision (CI not estimable in primary model), and one for indirectness due to varying diagnostic strategies across studies.
Major bleeding Follow-up: 90 days	OR 0.60 (0.40–0.89)	7 per 10,000 (to be updated)	96963 (5 studies)	⊕⊕⊕◯ Moderate	Not downgraded for inconsistency (I^2^ = 0%). Downgraded one level for imprecision (95% CI lower limit 0.40, suggesting potential benefit, but the interval remains wide).
Minor bleeding Follow-up: 90 days	OR 0.57 (0.49–0.66)	43 per 10,000 (to be updated)	76739 (4 studies)	⊕⊕◯◯ Low	Downgraded two levels: one for risk of bias (some studies were open-label), and one for serious inconsistency (moderate heterogeneity, I^2^ = 48%). Not downgraded for imprecision (narrow CI clearly suggesting benefit).

a The risk in the enoxaparin group is based on the median event rate across control groups in the included studies. The risk in the aspirin group (and its 95% confidence interval) is based on the assumed risk in the enoxaparin group and the relative effect of the intervention (and its 95% CI). The absolute effect calculations are pending update based on the new pooled estimates and should be revised accordingly.

CI, confidence interval; OR, odds ratio. GRADE Working Group grades of evidence: High quality (⊕ ⊕ ⊕⊕): We are very confident that the true effect lies close to that of the estimated effect. Moderate quality (⊕ ⊕ ⊕◯): We are moderately confident in the effect estimate: The true effect is likely to be close to the estimate of the effect, but there is a possibility that it is substantially different. Low quality (⊕ ⊕ ◯◯): Our confidence in the effect estimate is limited: The true effect may be substantially different from the estimate of the effect. Very low quality (⊕◯◯◯): We have very little confidence in the effect estimate: The true effect is likely to be substantially different from the estimated effect.

## 4 Discussion

### 4.1 Summary of evidence

This meta-analysis found no significant difference in the efficacy of aspirin vs. enoxaparin for the prevention of PE (OR = 1.14, 95% CI: 0.66–1.95), a finding consistent with several recent studies. A large randomized controlled trial (the METRC study) by O’Toole et al. (21) reported that aspirin was non-inferior to low-molecular-weight heparin (enoxaparin) in preventing 90-day all-cause mortality in patients undergoing surgery for extremity fractures, with mortality rates of 0.78% vs. 0.73%, respectively (absolute difference 0.05%, *p* < 0.001). Notably, the incidence of PE was identical between the two groups in that study (21), supporting our conclusion that there was no difference in PE prevention.

Regarding DVT prevention, our primary analysis showed no significant difference between the two agents (OR = 1.13, 95% CI: 0.65–1.98, *p* = 0.67). However, after excluding a study with high heterogeneity (6) in a sensitivity analysis, the incidence of DVT was significantly lower in the enoxaparin group compared to the aspirin group (OR = 0.78, 95% CI: 0.64–0.96, *p* = 0.02). This result aligns closely with the findings of the CRISTAL study group cluster-randomized trial by Sidhu et al. (11), which demonstrated a significantly higher rate of symptomatic VTE in the aspirin group than in the enoxaparin group (absolute difference 1.97%, *p* = 0.007) (6, 10). A potential explanation for this discrepancy lies in the distribution of DVT types; the CRISTAL study group trial observed that aspirin was less effective in preventing distal DVT (10), which accounted for 68% of the VTE events. In our meta-analysis, inconsistent anatomical classification of DVT across the included studies likely contributed to the high heterogeneity (I^2^ = 82%) observed in the primary analysis. It is important to note that patient population characteristics may significantly influence the outcomes. A 2022 study on patients with splenic rupture combined with lower extremity fractures found no significant difference in VTE rates between aspirin monotherapy and LMWH monotherapy (22). Conversely, a 2023 meta-analysis indicated that body mass index (BMI) did not significantly modify the comparative effectiveness of aspirin and enoxaparin (23), suggesting that the efficacy comparison might vary across different risk-stratified patient subgroups.

This study identified a significant advantage of aspirin regarding minor bleeding events (OR = 0.57, 95% CI: 0.49–0.66, *p* < 0.00001), while no significant differences were observed compared to enoxaparin in terms of major bleeding (OR = 0.60, 95% CI: 0.40–0.89, *p* = 0.01), wound complications (OR = 1.03, 95% CI: 0.60–1.78, *p* = 0.90), or mortality (OR = 0.81, 95% CI: 0.37–1.78, *p* = 0.60). This safety profile is consistent with prior research. The METRC study reported no significant difference in major bleeding events between aspirin and enoxaparin (21), and the CRISTAL study group trial similarly found comparable rates of major bleeding between the two groups.

Notably, the 43% reduction in the risk of minor bleeding (OR = 0.57) associated with aspirin observed in our study holds significant clinical implications. Although minor bleeding events are not directly life-threatening, they can substantially impact patient quality of life, medication adherence, and healthcare resource utilization. The mechanistic basis for this safety difference likely stems from the distinct anticoagulant mechanisms of the two drugs (22). Enoxaparin exerts a potent anticoagulant effect by inhibiting activated factor X (FXa), whereas aspirin produces a relatively milder antithrombotic effect by inhibiting platelet cyclooxygenase-1 (COX-1), thereby reducing thromboxane A2 generation (24–28). This difference in the mechanism of action also explains why enoxaparin demonstrates stronger efficacy in thromboprophylaxis but is associated with a higher bleeding risk.

Combining therapies may be a strategy to balance these effects. A 2022 study indicated (22) that the VTE incidence in the LMWH plus aspirin combination group (5.26%) was lower than in either the aspirin monotherapy (13.33%) or LMWH monotherapy (12.50%) groups, without increasing the bleeding risk. This suggests that a combination regimen might achieve an optimal benefit-to-risk balance for high-risk patients.

In summary, based on moderate-quality evidence, there is no significant difference between aspirin and enoxaparin in preventing pulmonary embolism or major bleeding. However, the superiority of enoxaparin in reducing DVT incidence is supported by only low-quality evidence, and the significant reduction in minor bleeding with aspirin is supported by moderate-quality evidence.

### 4.2 Clinical guideline recommendations

Our findings provide empirical support for the inclusion of aspirin as a viable prophylactic option in clinical guidelines. The comparable efficacy of aspirin and enoxaparin in preventing pulmonary embolism and major bleeding in our analysis, coupled with aspirin’s superior safety profile regarding minor bleeding, aligns with the recommendations of guidelines such as those from the American Academy of Orthopedic Surgeons (AAOS) and the American Society of Hematology (ASH), which endorse or suggest aspirin as an option for patients undergoing major orthopedic surgery, particularly those at standard or low risk for VTE (2, 11). Conversely, our sensitivity analysis suggesting a potential superiority of enoxaparin in preventing deep vein thrombosis supports the rationale for preferring LMWH in guidelines for patients identified as high-risk for VTE (e.g., those with a prior VTE history, active cancer, or thrombophilia).

Building upon this personalized paradigm, a hybrid or step-down strategy has emerged to further optimize the risk–benefit balance, particularly for patients at moderate to high risk. This approach involves initiating prophylaxis with a potent anticoagulant (e.g., enoxaparin or a direct oral anticoagulant) during the immediate high-risk postoperative period, followed by a transition to aspirin for the remainder of the prophylactic course. This strategy leverages the temporal decline in VTE risk, to provide strong early protection while limiting exposure duration to agents with higher bleeding potential. Supporting evidence for this model includes a study demonstrating the efficacy of risk-stratified prophylaxis using low-dose aspirin (29), and a recent large-scale evaluation confirming that a hybrid anticoagulant-to-aspirin regimen was associated with lower composite complication rates than aspirin monotherapy for patients at elevated VTE risk within an institutional risk-stratified framework (30).

Therefore, our meta-analysis contributes to the evolving evidence base for personalized prophylaxis: aspirin represents an effective and safer oral alternative for standard-risk patients, while a risk-stratified approach that may include initial anticoagulation (with or without subsequent transition to aspirin) remains crucial for those at elevated thrombotic risk.

### 4.3 Comparison with previous meta-analyses

Compared with previous important meta-analyses in this field, the present study demonstrates significant advances in the timeliness of included literature, sample size, and precision of the conclusions. The review by Nadi et al. (31) found no statistically significant differences between aspirin and enoxaparin across all thrombotic and bleeding outcomes but emphasized that the quality of evidence was “very low,” resulting in substantial uncertainty. Similarly, Farey et al. (32) found no significant differences; however, their analysis was limited by including only 4 RCTs (*n* = 1,507), which constrained statistical power and precluded definitive conclusions for key outcomes such as pulmonary embolism and mortality, particularly expressing caution regarding the safety profile in total hip arthroplasty patients.

Notably, two recent 2024 studies published after our literature search cut-off further inform this discussion. The network meta-analysis by Cheok et al. (33), which specifically evaluated thromboprophylaxis following hip and knee arthroplasty, concluded that aspirin (<325 mg daily), enoxaparin, and dabigatran offer an overall satisfactory efficacy and safety profile, strongly supporting the non-inferiority of aspirin in this context. Additionally, the systematic review by Williamson et al. (34), while focused on hip fracture surgery, found aspirin to be non-inferior to other agents for VTE prevention and potentially associated with reduced mortality, adding to the broader evidence for aspirin’s utility in orthopedic thromboprophylaxis.

Building on this evolving evidence base, our study incorporates 8 studies published through 2025, encompassing a total of 117,367 patients, thereby substantially enhancing statistical power. It not only confirms the non-inferiority of aspirin in preventing pulmonary embolism but, more importantly, through sensitivity analysis that eliminated heterogeneity interference, it is the first to clearly demonstrate the superiority of enoxaparin over aspirin in reducing the incidence of deep vein thrombosis (OR = 0.78, 95% CI: 0.64–0.96, *p* = 0.02). Concurrently, it establishes with highly significant evidence the definite advantage of aspirin in reducing the risk of minor bleeding (OR = 0.57, *p* < 0.00001).

Therefore, while building on the general framework of previous research and aligning with recent high-quality analyses, this study utilizes more recent and extensive data to provide more precise quantitative evidence the risk–benefit profiles of the two agents, offering stronger guidance for clinical decision-making.

### 4.4 Study limitations

This study has several limitations. First, significant heterogeneity was observed among the included studies, attributable to clinical variations in patient populations, prophylactic regimens, and methodology. Notably, the dosage and duration of prophylaxis varied substantially (e.g., aspirin 81–650 mg/day; enoxaparin 30 mg twice daily to 40 mg once daily), and the definitions for VTE diagnosis were inconsistent (systematic screening vs. clinical diagnosis). These sources of clinical and methodological heterogeneity, particularly the inconsistent diagnostic criteria, influenced the incidence of DVT and contributed to the statistical heterogeneity observed in our analyses. A planned subgroup analysis by surgical type (THA vs. TKA) was also not feasible due to the lack of procedure-specific data in most studies.

Second, the wide variability in prophylactic regimens is a key constraint on interpreting our pooled results. The anti-thrombotic effect and bleeding risk of these agents are dose-dependent. Consequently, the summary effect estimates should be interpreted as an average across a spectrum of clinically used doses rather than reflecting the effect of a specific, optimized regimen. Our inability to perform a meaningful subgroup analysis by dose, due to the limited number of studies within each dosing category, precludes conclusions about which specific dosing strategy optimizes the benefit–risk profile for either agent.

Third, the follow-up duration was primarily 90 days, which may not capture later complications. Furthermore, subgroup data for specific high-risk populations (e.g., those with extreme obesity or prior VTE) were insufficient, limiting the analysis of treatment effects in these cohorts.

Fourth, the generalizability of our findings may be limited by the geographic and economic context of the included evidence. The majority of studies were conducted in high-income countries. Differences in patient factors, healthcare systems, and socioeconomic realities may affect the applicability of these results to other settings, such as in Asia or in low-resource hospitals, where drug adherence patterns and healthcare access differ.

Fifth, the exclusion criteria for important modifiers of drug metabolism—such as renal impairment and obesity—varied across studies. This variability may influence the applicability of our findings to patient subgroups in whom drug dosing or pharmacokinetics are altered.

### 4.5 Future research directions

The favorable clinical profile of aspirin also suggests potential economic advantages. The significant reduction in minor bleeding events likely translates into lower costs associated with managing these complications (e.g., fewer dressings, clinic visits, or patient evaluations). Furthermore, oral administration of aspirin eliminates the need for syringes, nursing time for education and administration, and potential complications associated with subcutaneous injections, which are required for enoxaparin. This convenience may also improve adherence in the outpatient setting. When combined with aspirin’s inherently lower drug acquisition cost, these factors strongly indicate its superior cost-effectiveness for VTE prophylaxis in standard-risk patients undergoing major orthopedic surgery. This economic benefit further solidifies aspirin’s role as a valuable prophylactic option, particularly in resource-conscious healthcare systems.

## 5 Conclusion

This meta-analysis provides a nuanced comparison of aspirin and enoxaparin for VTE prophylaxis. Based on primary analyses, aspirin demonstrates comparable efficacy to enoxaparin in preventing overall VTE outcomes. However, sensitivity analyses adjusting for heterogeneity suggest that enoxaparin may be more effective in reducing deep vein thrombosis incidence, whereas aspirin is associated with a significantly lower risk of both major and minor bleeding events. Aspirin represents an effective and safer prophylactic option for patients in whom bleeding risk is a predominant concern, particularly given its oral administration and lower burden of minor bleeding. For patients at very high risk of thrombosis—especially distal DVT—the potential efficacy advantage of enoxaparin may justify its use, despite the associated increase in bleeding risk. Clinical decision-making should therefore be individualized, integrating patient-specific thrombotic and bleeding risks, as well as practical considerations such as route of administration and cost. Therefore, aspirin may be a preferable option in settings where safety, convenience, and resource utilization are prioritized.

## Data Availability

The raw data supporting the conclusions of this article will be made available by the authors, without undue reservation.

## Footnotes

1
https://www.cochrane.org/
